# A structural equation model of perceived and internalized stigma, depression, and suicidal status among people living with HIV/AIDS

**DOI:** 10.1186/s12889-018-5053-1

**Published:** 2018-01-15

**Authors:** Chengbo Zeng, Linghua Li, Yan Alicia Hong, Hanxi Zhang, Andrew Walker Babbitt, Cong Liu, Lixia Li, Jiaying Qiao, Yan Guo, Weiping Cai

**Affiliations:** 1 Department of Biostatistics and Epidemiology, School of Public Health, Sun Yat-sen University, #74 Zhongshan 2nd Road, Guangzhou, 510080 China; 2 Department of Infectious Disease, Guangzhou Number Eight People’s Hospital, #627 Dongfeng Road, Guangzhou, 510080 China; 3 Department of Health Promotion and Community Health Sciences, School of Public Health, Health Science Center, Texas A&M University, College Station, TX 77843 USA; 4 Department of Epidemiology and Health Statistics, School of Public Health, Guangdong Pharmaceutical University, #283 Jianghai Street, Guangzhou, 510220 China; 5 Sun Yat-sen Center for Migrant Health Policy, Sun Yat-sen University, Guangzhou, China; 6 Sun Yat-sen Global Health Institute, Institute of State Governance, Sun Yat-sen University, Guangzhou, China

**Keywords:** Stigma, Depression, Suicidal status, People living with HIV/AIDS (PLWH), China

## Abstract

**Background:**

Previous studies have shown positive association between HIV-related stigma and depression, suicidal ideation, and suicidal attempt among people living with HIV/AIDS (PLWH). But few studies have examined the mechanisms among HIV-related stigma, depression, and suicidal status (suicidal ideation and/or suicidal attempt) in PLWH. The current study examined the relationships among perceived and internalized stigma (PIS), depression, and suicidal status among PLWH in Guangzhou, China using structural equation modeling.

**Methods:**

Cross-sectional study by convenience sampling was conducted and 411 PLWH were recruited from the Number Eight People’s Hospital from March to June, 2013 in Guangzhou, China. Participants were interviewed on their PIS, depressive symptoms, suicidal status, and socio-demographic characteristics. PLWH who had had suicidal ideation and suicidal attempts since HIV diagnosis were considered to be suicidal. Structural equation model was performed to examine the direct and indirect associations of PIS and suicidal status. Indicators to evaluate goodness of fit of the structural equation model included *Chi-square Statistic*, *Comparative Fit Index* (*CFI*), *Root Mean Square Error of Approximation* (*RMSEA), Standardized Root Mean Square Residual* (*SRMR*)*,* and *Weighted Root Mean Square Residual* (*WRMR*).

**Results:**

More than one-third (38.4%) of the PLWH had depressive symptoms and 32.4% reported suicidal ideation and/or attempt since HIV diagnosis. The global model showed good model fit (*Chi-square value* = 34.42, *CFI* = 0.98, *RMSEA* = 0.03, *WRMR* = 0.73). Structural equation model revealed that direct pathway of PIS on suicidal status was significant (standardized pathway coefficient = 0.21), and indirect pathway of PIS on suicidal status via depression was also significant (standardized pathway coefficient = 0.24). There was a partial mediating effect of depression in the association between PIS and suicidal status.

**Conclusions:**

Our findings suggest that PIS is associated with increased depression and the likelihood of suicidal status. Depression is in turn positively associated with suicidal status and plays a mediating role between PIS and suicidal status. Therefore, to reduce suicidal ideation and attempt in PLWH, targeted interventions to reduce PIS and improve mental health status of PLWH are warranted.

## Background

With the availability of antiretroviral therapy (ART), the morbidity and mortality of people living with Human immunodeficiency virus (HIV)/Acquired immune deficiency syndrome (AIDS) have been decreasing, and the number of people living with HIV/AIDS (PLWH) has been increasing in China [1]. According to China’s AIDS Response Progress Report, reported cases of PLWH continued to increase from 307,000 in 2010 to 501,000 in 2014 [1]. Although the life expectancy among PLWH has increased due to ART, many other challenges to health persist, including challenges such as opportunistic infections, syphilis, tuberculosis, and prevalent HIV-related stigma [1, 2].

Stigma was traditionally defined as “a significantly discrediting trait” and further expanded as “a powerful discrediting and tainting social label that radically changes the way individuals view themselves and are viewed as persons” [3, 4]. People who have been diagnosed with HIV/AIDS may be stigmatized, because the routes of HIV infection are usually controllable and HIV-related risk behaviors (e.g., unprotected sexual behavior with multiple or same-sex partners, injection drug use) are not socially accepted in China [2]. In addition, since HIV/AIDS is characterized as a degenerative and unalterable illness, people may have fears (e.g., fear of the disease, contagion, and death) towards PLWH and such fears may lead to universal expression of stigma [5]. On the other hand, PLWH who view themselves as persons responsible for their own behaviors are likely to experience intensive shame and guilt towards themselves [6].

The prevalent stigma PLWH are likely facing in China may be further explained by the collective culture and perspective of traditional Chinese medicine [7]. Collective culture in China demands that individuals’ behaviors conform to social norms and punish those whose behaviors are deviant [7–9]. As some PLWH engage in HIV-related risk behaviors (e.g., commercial sex, homosexuality, and intravenous drug use) deviant from the socially approved norms, PLWH as a group are faced with social disapproval and discrimination [7]. On the other hand, traditional Chinese medicine often associates a disease “caused by immoral behaviors” with “a spiritual attack from evil sources that have lodged in the body or taken over the person” [7]. Thus, PLWH are considered to be associated with evil spirit and stigmatized from the perspective of traditional Chinese medicine.

Perceived and internalized stigma (PIS) includes two important types of stigma, which are prevalent among PLWH and closely related to mental and behavioral health [10, 11]. Perceived HIV-related stigma is defined as awareness of discriminatory and prejudicial attitudes from people in the society [12]. People who internalize stigma have negative beliefs and self-images and often low self-esteem as a result of internalizing negative views from the society [10, 12, 13]. Individuals who perceive stigma from other people in the society are usually vulnerable to feelings of self-hatred especially when they internalize the negative views of themselves from the society [10]. The combined effects of perceived and internalized stigma may lead to a series of consequences, such as non-disclosure of HIV infection, seclusion, depressive symptoms, and suicidal ideation and attempt [11, 14].

Literature has shown that perceived and internalized stigma plays a critical and direct role on depression [10, 11]. A cross-sectional study of 310 female sex workers (FSWs) in Guangxi, China reported that perceived stigma was significantly associated with FSWs’ poor mental health (e.g., elevated depressive symptoms, suicidal ideation, and suicidal attempt) [11]. Another study of 268 PLWH in Milwaukee, Madison, and New York City found that internalized stigma was significantly associated with depression, anxiety, and hopelessness [10].

Consequences of depression in PLWH are many, such as weakening treatment effects, accelerating progression of AIDS, deteriorating immune system, increasing risks of morbidity and mortality, and decreasing quality of life of PLWH [15–18]. One of the most detrimental consequences of depression in PLWH is the increased likelihood of committing suicide in this population, as many studies found that PLWH reported elevated levels of suicide [19–21]. According to O’Carrol, suicidal status is defined as self-reported suicidal ideation and/or suicidal attempt, in which suicidal ideation is the consideration of committing suicide, and suicidal attempt is the actual action of committing suicide [22, 23].

Most studies on suicidal status in PLWH were conducted in the western countries, with few studies reported from developing countries such as China [19–21, 24–27]. Among the few, one study, conducted among 184 HIV positive men who have sex with men (MSM) in Anhui province of China reported that 31% and 5.4% of the participants had had suicidal ideation and suicidal attempt respectively in the past six months. The previous study also found that both perceived stigma and depression were significantly associated with increased likelihood of suicidal ideation in HIV-seropositive MSM [24]. Existing studies on stigma, depression, and suicide among PLWH have typically employed relational analyses demonstrating associations between these concepts, few have examined the mediating effect and mechanisms among these concepts.

The current study will employ structural equation model (SEM) to explore the mediating effect of depression and mechanisms among PIS, depression, and suicidal status in PLWH in addition to examining the proportions of depression and suicidal status among PLWH. We hypothesize that: (1) PIS has a significant direct effect on both depression and suicidal status; and (2) PIS has a significant indirect effect on suicidal status, mediated by depression (a higher level of PIS is associated with a higher level of depression, which in turn is associated with increased likelihood of suicidal status).

## Methods

### Study site

Participants were recruited from outpatient and inpatient departments of an HIV/AIDS treatment hospital in Guangzhou, China in 2013. Guangzhou, the capital city of Guangdong province, is the third biggest city in China and the biggest in South China, with 8.54 millions resident population in 2015 [28]. The hospital is the only treatment provider for PLWH in the metropolitan area of Guangzhou.

### Participants and sampling

A cross-sectional study by convenience sampling was conducted. Inclusion criteria for the current study were HIV-seropositive status (registered in the hospital system or with an official document), at least 18 years of age (self-reported and verified by the official document when needed), willing to provide written informed consent, and agreeing to participate in the study. PLWH who reported unable to finish the questionnaire due to mental illness or other reasons (e.g., not having had enough time) were excluded. Participants were recruited through direct approach of our outreach staff. PLWH would be recruited if they agreed to provide written informed consent. Patients who met the inclusion criteria were asked to complete a paper-based questionnaire in the waiting room with an interviewer being present. The interviewer would only provide assistance upon request. Interviewers had received extensive training on research ethics and assessment methodology prior to data collection. A meal voucher or a small gift equivalent to two US dollars was given to the participants as a token of appreciation for their participation. A total of 450 PLWH were recruited and 39 questionnaires were invalid as participants did not finish these questionnaires due to various reasons (e.g., physical examination, outpatient appointment), resulting in 411 (91.3%, 411/450) PLWH in the current study. The current study was approved by the Institutional Review Board of Sun Yat-sen University.

### Measurements

#### Socio-demographic characteristics

Participants provided socio-demographic characteristics including age (in years), gender (1 = male, 0 = female), ethnicity (1 = Han, 0 = others), education (1: <high school, 2: high school, 3: >high school), marital status (1 = never married, 2 = married/cohabited, 3 = separated/divorced/widowed), sexual orientation (1 = heterosexual, 0 = homosexual/bisexual/uncertain), and duration since HIV diagnosis (in years).

#### Perceived and internalized stigma

Perceived and internalized stigma (PIS) was measured by fourteen statements derived from HIV Stigma Scale [29, 30]. The fourteen statements of PIS are presented in Table 1. Validity and reliability of both the original HIV Stigma Scale (Cronbach’s alpha = 0.95) and the 14-item version used in the current study (Cronbach’s alpha> 0.90) have been validated and established in China [29, 31]. Cronbach’s alpha of PIS for the current study was 0.93. PIS scale measures two dimensions of stigma, including perceived stigma and internalized stigma [29]. Statements were evaluated using a 4-point Likert-type scale (*strongly disagree, disagree, agree, and strongly agree*), with higher scores indicating higher levels of stigma. Total score of the scale ranged from 14 to 56, and subtotal scores of perceived stigma and internalized stigma ranged from 6 to 24 and 8 to 32, respectively. To explore the proportion of different levels of PIS among PLWH, we categorized PIS into three levels: low (scale score = 14–28), medium (scale score = 29–42), and high (scale score = 43–56) level [11]. In SEM modelling, PIS was measured by the two subscales (perceived stigma and internalized stigma) that were treated as continuous subscale scores.

**Table 1:** Perceived and internalized stigma scale

Items of the scale	Domains
1. I feel guilty because I have HIV.	Internalized stigma
2. People’s attitudes about HIV make me feel worse about myself.	Internalized stigma
3. People with HIV lose their jobs when their employers learn.	Perceived stigma
4. I feel I am not as good a person as others because I have HIV.	Internalized stigma
5. People with HIV are treated like outcasts.	Perceived stigma
6. Most people think that a person who has HIV is dirty.	Perceived stigma
7. It is easier to avoid new friendships than worry about telling someone that I have HIV.	Internalized stigma
8. Having HIV makes me feel unclean.	Internalized stigma
9. Since learning I have HIV, I feel set apart and isolated from the rest of the world.	Internalized stigma
10. Most people think that a person with HIV is disgusting.	Perceived stigma
11. Having HIV makes me feel that I am a bad person.	Internalized stigma
12. Most people with HIV are rejected when others find out.	Perceived stigma
13. Most people are uncomfortable around someone with HIV.	Perceived stigma
14. Having HIV in my body is disgusting to me.	Internalized stigma

#### Depressive symptoms

Depressive symptoms were measured by the Chinese version of the Center for Epidemiologic Studies Depression Scale (CES-D), a 20-item scale with four dimensions, including *depressed affect*, *positive affect*, *somatic and retarded activity*, and *interpersonal problems* [32, 33]. Of the four dimensions, only positive affect was reverse-coded. Validity and reliability of the scale have been validated and established in China [33]. Participants were asked during the past week whether they had felt or behaved as the following (e.g., “bothered by things that don’t usually bother me”, “did not feel like eating”). Items were scored from zero (rarely or none = less than 1 day) to three (most or all of the time = 5–7 days), with a higher score indicating a greater level of depression. The total score of the scale ranged from 0 to 60. PLWH with CES-D scores no less than 16 were considered as having had depressive symptoms. Internal consistency estimate of reliability of the scale was good (Cronbach’s alpha = 0.93). In SEM modelling, depression was measured by the four constructs (depressed affect, positive affect, somatic and retarded activity, and interpersonal problems) with each treated as a continuous subscale score.

#### Suicidal status

As the number of PLWH who had attempted suicide since HIV diagnosis was small (37, 9%), suicidal ideation and suicidal attempt were combined into one variable of suicidal status in the current study [23]. Participants were asked whether they had ever seriously considered committing suicide since HIV diagnosis (yes/no) and whether they had ever actually attempted suicide since HIV diagnosis (yes/no) [11]. Participants who answered “yes” to the first question were considered to have had suicidal ideation, and “yes” to the second question were considered to have had suicidal attempt [11]. Those who answered “yes” to either of the two questions (suicidal ideation or attempt) were considered having had suicidal status.

#### Data analysis

First, missing values were addressed by using multiple imputation in the current study. Descriptive statistics were reported on perceived stigma, internalized stigma, depression, suicidal ideation, suicidal attempt, and socio-demographic characteristics (e.g., age, gender, marital status). Mean ± standard deviation (*SD*) were used to describe normally-distributed continuous variables, median (interquartile range, *IQR*) for skewedly-distributed continuous variables, and frequencies (percentages) for categorical variables.

Second, bivariate analyses were conducted to examine the relationships between socio-demographic and HIV-related characteristics and suicidal status. Independent-samples *t* tests were used to examine the relationships between normally-distributed variables (i.e., age) and suicidal status, *Wilcoxon rank-sum* tests for the relationships between skewedly-distributed variables (i.e., perceived stigma, internalized stigma, and depression) and suicidal status, and *Chi-square* tests for categorical variables (i.e., marital status). *Spearman* correlation analyses were performed to examine the associations among key continuous variables (perceived stigma, internalized stigma, and depression).

Third, since the factors of the latent variables (i.e., PIS and depression) had been established in the previous studies, both PIS and depression were measured by their subscales in structural equation modeling [34]. In the measurement model of PIS and depression, mean scores of domains of PIS and depression were considered as indicators of the variables (e.g., the average score of internalized stigma and perceived stigma as indicators of PIS). Confirmatory factor analysis (CFA) was performed to assess the goodness of fit of the measurement model. With a satisfactory measurement model, structural equation model (SEM) was then performed to assess the hypothesized relationships among PIS, depression, and suicidal status. Socio-demographic characteristics that were significantly associated with suicidal status in bivariate analyses were controlled as covariates in the SEM.

In the SEM, direct effect of PIS on suicidal status and mediating effect of depression on the association between PIS and suicidal status were examined. Bias-corrected bootstrap procedure based on 1000 bootstrap samples was employed to test the mediating effect [35]. According to Shrout and Bolger, bias-corrected confidence intervals for direct and indirect pathways were reported [36]. Wald *Chi-square* test was performed to examine the difference between direct and indirect effect (mediating effect).

Indicators to evaluate goodness of fit of measurement and structural models in the current study included *Chi-square Statistic*, *Comparative Fit Index* (*CFI*), *Root Mean Square Error of Approximation* (*RMSEA*), *Standardized Root Mean Square Residual* (*SRMR*), and *Weighted Root Mean Square Residual* (*WRMR*) [37]. Smaller *Chi-square values* indicate better model fit. *CFI* ≥ 0.95, *RMSEA* ≤ 0.06, *SRMR* ≤ 0.08, and *WRMR* ≤ 1.00 indicate good model fit [37]. *P* < 0.05 is considered to be statistically significant.

Descriptive statistics, correlation analyses, and bivariate analyses were performed using SAS software version 9.4 (SAS Institute, Inc., Cary, NC, U.S.). CFA and SEM were performed using Mplus Version 7.0 (Muthen & Muthen, Los Angeles, CA, U.S.). CFA was tested by robust maximum likelihood method (estimator = MLR in Mplus). SEM was tested by robust weighted least squares (WLS) approach (estimator = WLSMV in Mplus).

## Results

### Descriptive statistics

As shown in Table 2, the average age was 39.0 (±9.1) years with a range from 20 to 76; 69.6% were male and 30.4% were female. The majority of the participants were Han ethnicity (93.4%). More than half of the participants were married or cohabited (52.8%), and had received no more than high school education (54.5%). About two-thirds (68.1%) of the participants were heterosexual and one-third (31.9%) homosexual or bisexual or uncertain. The median years of HIV infection since diagnosis was 2.7 years (*IQR*: 1.1–5.0). Medians (*IQR*) of the scores of PIS, perceived stigma, internalized stigma, and depression were 35 (29–41), 17 (14–19), 18 (14–22), and 13 (10–19), respectively. The majority of the participants had a medium level of PIS (63.8%), followed by 22.4% of a low level and 13.9% of a high level. With 16 being the cut-off point, 38.4% of the PLWH reported elevated depressive symptoms. About one-third (29.7%) of the participants reported having seriously considered committing suicide since HIV diagnosis, and 9.0% reported having attempted suicide since HIV diagnosis; altogether 32.4% of the participants had had suicidal ideation and/or attempt since HIV diagnosis.

**Table 2:** Descriptive statistics among PLWH in Guangzhou, China (*n* = 411)

Characteristics	Total (*%*)	Characteristics	Total (*%*)
Age (years; *mean* ± *SD*)	39.0 ± 9.1	Marital status	
Gender		Never married	138 (33.6)
Male	286 (69.6)	Married/cohabited	217 (52.8)
Female	125 (30.4)	Separated/divorced/widowed	56 (13.6)
Ethnicity		Sexual orientation	
Han	384 (93.4)	Heterosexual	280 (68.1)
Others	27 (6.6)	Homosexual/bisexual/uncertain	131 (31.9)
Education		Duration of HIV infection (years, Median (IQR))	2.7 (1.1, 5.0)
< High school	224 (54.5)	Perceived stigma (Median (IQR))	17 (14, 19)
High school	88 (21.4)	Internalized stigma (Median (IQR))	18 (14, 22)
> High school	99 (24.1)	PIS (Median (IQR))	35 (29, 41)
Suicidal ideation		Low	92 (22.4)
Yes	124 (29.7)	Medium	262 (63.8)
No	282 (70.3)	High	57 (13.9)
Suicidal attempt		Depression (Median (IQR))	13 (10, 19)
Yes	37 (9.0)	Depressive symptoms	
No	374 (91.0)	Yes	158 (38.4)
Suicidal status		No	253 (61.6)
Yes	133 (32.4)	–	–
No	273 (67.6)	–	–

*n* Sample size, PIS Perceived and internalized stigma, *SD* Standard deviation, *IQR* Interquartile range

### Correlations between demographic and HIV-related characteristics and suicidal status

Table 3 shows the associations between demographic and HIV-related characteristics and suicidal status. Bivariate analyses indicated that gender and sexual orientation were significantly associated with suicidal status among PLWH. Specifically, males were more likely to have had suicidal ideation and/or attempt than females (35.7% vs. 24.8%, *p* < 0.05) since HIV diagnosis. There were a higher proportion of PLWH who were homosexual/bisexual/uncertain to have had suicide status than those heterosexual (42.0% vs. 27.9%, *p* < 0.01). In addition, perceived stigma, internalized stigma, and depression were also significantly associated with suicidal status among PLWH (*p* < 0.01).

**Table 3:** Bivariate analysis of suicidal status among PLWH in Guangzhou, China (*n* = 411)

Characteristics (*n*)	Total (*%*)	Suicidal status (*%*)		*P-value*
		Yes	No	
Sample size	411	133 (32.4)	273 (67.6)	–
Age (years; *mean* ± *SD*)	39.0 ± 9.1	38.2 ± 9.1	39.4 ± 9.1	0.23^a^
Gender				0.03^*b^
Male	286 (69.6)	102 (35.7)	184 (64.3)	
Female	125 (30.4)	31 (24.8)	94 (75.2)	
Ethnicity				0.59^b^
Han	384 (93.4)	123 (32.0)	261 (68.0)	
Others	27 (6.6)	10 (37.0)	17 (63.0)	
Education				0.63^b^
< High school	224 (54.5)	68 (30.4)	156 (69.6)	
High school	88 (21.4)	31 (35.2)	57 (64.8)	
> High school	99 (24.1)	34 (34.3)	65 (65.7)	
Marital status				0.40^c^
Never married	138 (33.6)	50 (36.2)	88 (67.8)	
Married/cohabited	217 (52.8)	64 (29.5)	153 (70.5)	
Separated/divorced/widowed	56 (13.6)	19 (33.9)	37 (66.1)	
Sexual orientation				< 0.01^**c^
Heterosexual	280 (68.1)	78 (27.9)	202 (72.1)	
Homosexual/bisexual/uncertain	131 (31.9)	55 (42.0)	76 (58.0)	
Duration of HIV infection (years; Median (IQR))	2.7 (1.1, 5.0)	2.2 (0.9, 4.7)	2.8 (1.1, 5.0)	0.27^d^
Perceived stigma (Median (IQR))	17 (14, 19)	18 (16, 21)	17 (13, 19)	< 0.01^**d^
Internalized stigma (Median (IQR))	18 (14, 22)	21 (18, 24)	17 (13, 21)	< 0.01^**d^
PIS (Median (IQR))	35 (29, 41)	39 (33, 42)	33 (28, 39)	< 0.01^**d^
Depression (Median (IQR))	13 (10, 19)	17 (12, 23)	12 (9, 17)	< 0.01^**d^

*n* Sample size, PIS Perceived and internalized stigma, *SD* Standard deviation, *IQR* Interquartile range

*: *p* < 0.05; **: *p* < 0.01

^a^Independent-samples *t* test

^b^*Chi-square* test

^c^*Fisher’s* exact test

^d^*Wilcoxon rank-sum* test

### Correlations among perceived stigma, internalized stigma, and depression

Table 4 shows correlations among perceived stigma, internalized stigma, and depression. Results of correlation analyses indicated that both perceived stigma and internalized stigma were positively associated with depression, and their Spearman correlation coefficients were 0.22 (*p* < 0.01) and 0.39 (*p* < 0.01), respectively.

**Table 4:** Correlation coefficients matrix among perceived stigma, internalized stigma, and depression (*n* = 411)

Variables	Perceived stigma	Internalized stigma	Depression
Perceived stigma	1.00		
Internalized stigma	0.55**	1.00	
Depression	0.22**	0.39**	1.00

*n* Sample size

**: *p* < 0.01

### Measurement model

**Fig. 1:**
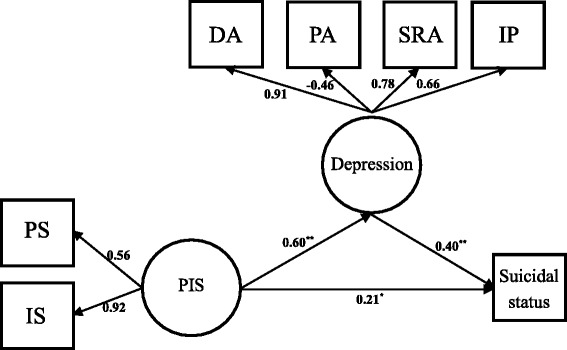
Measurement and structural model of PIS, depression, and suicidal status among PLWH (*n* = 411). All factor loadings were significant at *p* < 0.05 level. *: *p* < 0.05; **: *p* < 0.01. DA: mean score of depressed affect; PA: mean score of positive affect; SRA: mean score of somatic and retarded activity; IP: mean score of interpersonal problems; PS: mean score of perceived stigma; IS: mean score of internalized stigma. Gender and sexual orientation significantly associated with suicidal status in bivariate analysis were controlled as covariates in the SEM

CFA indicated that measurement model yielded a good model fit (*Chi-square value* = 22.55, *Degrees of Freedom* (*DF*) = 9, *p* < 0.01, *CFI* = 0.98, *RMSEA* = 0.06, *SRMR* = 0.03). All of the factor loadings were significant at *p* < 0.01 level. Results of the standardized factor loadings are shown in Fig. 1.

### Structural model

Controlling for gender and sexual orientation that were significantly associated with suicidal status in bivariate analyses, results of SEM indicated that the overall model had a satisfactory model fit (*Chi-square value* = 34.42, *DF* = 25, *p* = 0.10, *CFI* = 0.98, *RMSEA* = 0.03, *WRMR* = 0.73).

### Direct and indirect effects of structural model

Direct pathway from PIS to suicidal status was significant (standardized pathway coefficient = 0.21, *p* = 0.01), and indirect pathway from PIS to suicidal status was also significant (standardized pathway coefficient = 0.60 × 0.40 = 0.24, *p* < 0.01). The results indicated that there was a partial mediating effect of depression and that the standardized effect of indirect pathway was comparable to that of the direct pathway (0.24 vs. 0.21) with no significant difference (*Wald Chi-square value* = 0.04, *p* = 0.85) (Tables 5 and 6). The final model of SEM is presented in Fig. 1.

**Table 5:** Pathway coefficients of structural equation model (*n* = 411)

Pathways	Estimate	Std. Estimate	95% CI	*SE*	*P*-value
PIS → Depression	0.54	0.60	0.45~ 0.64	0.05	< 0.01**
Depression→Suicidal status	0.60	0.40	0.37~ 0.82	0.11	< 0.01**
PIS → Suicidal status	0.29	0.21	0.06~ 0.52	0.12	0.01*

*n* Sample size, PIS Perceived and internalized stigma, *Std. Estimate* Standardized estimate, *CI* Confidence interval, *SE* Standard error

*: *p* < 0.05; **: *p* < 0.01

**Table 6:** Coefficients of measurement model and structural model among PLWH (*n* = 411)

Factor loadings	Estimate	Std. Estimate	95%CI	*SE*	*P-*value
Depression					
Depressed affect	1.00	0.91	–	–	–
Positive affect^a^	− 0.69	−0.46	− 0.86~ − 0.51	0.09	< 0.01**
Somatic and retarded activity	0.80	0.78	0.70~ 0.90	0.05	< 0.01**
Interpersonal problems	0.69	0.66	0.60~ 0.79	0.05	< 0.01**
PIS					
Internalized stigma	1.00	0.92	–	–	–
Perceived stigma	0.63	0.56	0.51~ 0.75	0.06	< 0.01**
Pathway effect	Estimate	Std. Estimate	95%CI	*SE*	*P-*value
Total effect	0.61	0.45	0.45~ 0.78	0.09	< 0.01**
Direct effect					
PIS → Suicidal status	0.29	0.21	0.06~ 0.52	0.12	0.01*
Indirect effect					
PIS → Depression→Suicidal status	0.32	0.24	0.20~ 0.45	0.07	< 0.01**

*n* Sample size, PIS Perceived and internalized stigma, *Std. Estimate* Standardized estimate; *CI* Confidence interval, *SE* Standard error

*: *p* < 0.05; **: *p* < 0.01

^a^reverse-coded

## Discussion

The current study examined the relationships among PIS, depression, and suicidal status of PLWH in Guangzhou, China. The results showed that PLWH suffered from relatively higher levels of PIS and some PLWH had perceptions of HIV-related discrimination and/or negative self-images of themselves with the majority (63.8%) of the participants having experienced a medium level of PIS and 13.9% a high level. 38.4% of the PLWH reported depressive symptoms. The proportion of depressive symptoms in the current study was lower than the median prevalence of depressive symptoms among the general PLWH in China (60.6%) from a systematic review [38]. However, the median prevalence of depressive symptoms among PLWH in the systematic review was based on studies mostly conducted in rural China with smaller sample sizes (< 200) while the current study was conducted in Guangzhou, the third biggest city in China, with a larger sample size (411) [38, 39]. PLWH in rural China often have more mental health problems than those in big cities [38]. Even though PLWH in the current study had a smaller proportion of depressive symptom than the general PLWH in China, they were more likely to suffer from depression than populations with other chronic diseases such as diabetes (e.g., 38.4% > 31.0%) [40]. As depression and suicidal status were closely linked, the PLWH also had a high proportion of suicidal status (32.4%). Proportions of suicidal ideation (29.7%) and suicidal attempts (9.0%) in the PLWH in the current study were notably higher than the lifetime rates of 3.1% and 1%, respectively, among the general metropolitan population in China [41]. The findings of rather high proportions of suicidal ideation and suicidal attempt in PLWH are of urgent public health concern.

Findings of high proportions of suicidal ideation (29.7%) and suicidal attempt (9.0%) among PLWH in the current study were similar to those of an earlier study of 184 HIV-seropositive MSM in Anhui province of China (suicidal ideation 31.0% and suicidal attempt 5.4%) [24]. Though the two studies had similar proportions of suicidal ideation, the Anhui study collected information on suicide 6 months prior to the study, whereas the present study collected suicidal information since HIV-seropositive diagnosis and the median duration since HIV diagnosis was 2.7 years. In addition, the participants in the current study were individuals who were HIV-seropositive, whereas in the previous study were MSM who were HIV-seropositive. Anhui province is located in the inland of China while Guangzhou is in the frontier of China’s economic reform. One major advantage of the current study compared to the previous study is that SEM allows investigation of mechanisms among PIS, depression, and suicidal status (e.g., direct and mediating effect).

The present study is the first effort to examine the intertwined relationships among PIS, depression, and suicidal status of PLWH in China. Results from the SEM revealed that PIS had both direct and indirect effects on suicidal status. People who reported higher levels of PIS were more likely to have seriously considered committing suicide and/or had suicidal attempts. Meanwhile, higher levels of PIS were also associated with higher levels of depression, which in turn were associated with increased likelihood of suicide. Thus, depression played a mediating role on the association between PIS and suicidal status.

The current study may shed light on the mechanism of how PIS affects the likelihood of suicide in PLWH. As HIV/AIDS is often regarded as a fatal infectious disease in China and associated with tremendous social disapproval and stigma, PLWH suffer from immense psychological and social burdens (e.g., social isolation) [2, 42]. High levels of stigma are associated with mental illness, especially depression, which is associated with suicidal ideation and suicidal attempt [10, 12, 19–21]. Studies have also indicated that high levels of stigma may reduce coping self-efficacy of PLWH, which may lead to increased suicidal ideation [43, 44]. A study of 2909 PLWH in the four US cities (San Francisco, Los Angeles, Milwaukee, and New York City) found that PLWH who reported lower levels of coping self-efficacy were more likely to report suicidal ideation [44]. The mediating role of depression on the association between stigma and suicide is confirmed in the current study, but the plausible explanation of coping self-efficacy needs further exploration in future research.

Since PIS is directly associated with both depression and suicidal status, reducing PIS is not only conducive to alleviate depressive symptoms in PLWH, but also important to reduce the likelihood of suicide in this population. The severity of suicidal status may well be underestimated in the current study, as those who had successfully committed suicide were excluded from the sample. Targeted interventions to reduce PIS are urgently needed for the psychological well-being of PLWH and reduction of suicide in PLWH.

In the efforts of preventing suicide in PLWH, it is of great importance and urgency to improve their mental health status, as depression may lead to suicidal ideation and attempt [21]. Results of bivariate analyses showed that PLWH who had higher level of depression were more likely to have suicidal ideation or suicidal attempt. In addition, outcomes of SEM showed that the impact of depression on suicidal status was significant. Thus, the risk of suicide would increase as the level of depression increased among PLWH. Furthermore, depression played a partial mediating role on the association between PIS and the likelihood of suicide, and the effect of indirect pathway was comparable to the effect of direct pathway, with no statistical significance between the two (*Wald Chi-square value* = 0.04, *p* = 0.85). The impact of PIS on suicide through this indirect path is as important as the direct path, which suggests that in order to reduce suicide of PLWH, targeted interventions to reduce depression in PLWH may be equally effective as to reduce the impact of PIS on suicidal status of PLWH. All the above evidences indicate that to improve mental health of PLWH is critical and potentially effective in reducing suicide in PLWH.

It is also worth mentioning the effects of gender and sexual orientation on suicidal status of PLWH. PLWH who were male were more likely to have suicidal ideation or attempt suicide than PLWH who were female (35.7% vs. 24.8%). PLWH who were homosexual/bisexual/uncertain had a much higher proportion of suicide than those who were heterosexual (42.0% vs. 27.9%). In the current study, almost all of the PLWH who were homosexual/bisexual/uncertain were male (97.7%). Among the PLWH who were homosexual/bisexual, two-thirds (66.4%) were never married. PLWH who were homosexual/bisexual were facing double stigma from both HIV infection and sexual orientation [24, 45–47]. In the traditional Chinese culture, getting married, bearing children, and carrying on family names are considered to be the most important responsibilities for men [45, 47]. Males who fail to fulfill these obligations are regarded as shameful, selfish, and shrinking from their familial responsibilities [45]. Suffering from double stigma and not being able to fulfill their familial responsibilities, PLWH who are homosexual/bisexual may experience tremendous stress and consequent mental illnesses (e.g., depression, anxiety). Differences in suicidal status between male and female, and between homosexual/bisexual and heterosexual in PLWH found in the current study deserve further investigation in the future.

Efficient and effective HIV prevention and intervention efforts to reduce suicide in PLWH should be designed and implemented at both societal and individual levels. At societal level, structured efforts should be made to promote public education of HIV-related knowledge to both the general population and healthcare providers, to improve awareness and reduce stigma towards HIV/AIDS and its carriers [48–51]. Policies should also be made at the societal level to enhance social support towards PLWH, to improve their psychological well-being (e.g., less depressive symptoms, less anxiety) and health-related behaviors (e.g., active coping, medication adherence, regular check-up). Policies should not only be made for the formal health care institutions (e.g., hospitals, centers for disease control and prevention (CDCs)), but also for non-governmental organizations (NGOs) that have played an important role in HIV prevention and patient care (e.g., health education, behavioral intervention, and care for PLWH) [52, 53]. Many NGOs are not officially registered and acknowledged in China even though some of them are working for the government (e.g., CDCs). In addition, financial investments on NGOs should be increased and engagement of NGOs to HIV/AIDS prevention and care for PLWH should be encouraged [52, 53]. At individual level, psychological counseling and interventions are needed to reduce individuals’ internalized stigma, depression, and suicidal ideation, and to improve mental health status and quality of life of PLWH [54–57]. However, such efforts, especially well-designed psychosocial programs, are still much needed for most PLWH in China [58–60].

There are some limitations in the current study. First, this is a cross-sectional study, thus causal relationships cannot be drawn from the study. To verify the mediating effect of depression on the association between PIS and suicidal status, longitudinal studies are needed. Second, as all measurements were self-reported, recall biases of some questions, such as depression and suicidal status (suicidal ideation and suicidal attempt), might exist. However, proportions of depression and suicide were likely to be more conservative in the current study due to response biases to sensitive questions and unavoidable sampling bias as those who had successfully committed suicide were excluded from the current study.

Third, the current study did not differentiate inpatient from outpatient PLWH who might be different in socio-demographic characteristics, depression, suicidal status, and PIS. Further research with larger samples of both inpatient and outpatient PLWH are needed to enable testing the equality of factor structures and structural relationships between the two groups. Fourth, results of the current study may have limited generalizability to PLWH who refused to participate in this study and who were not clinic-based as the latter might be more stigmatized and marginalized with elevated mental health problems. Finally, cautions should also be given to generalize the results from the current study in one metropolitan city to other areas of China or worldwide.

## Conclusions

In conclusion, the current study found that PLWH had experienced a high level of suicidal status (suicidal ideation and suicidal attempt). In the context of Chinese culture, the current study provided insights into the relationships among PIS, depression, and suicidal status in PLWH and evidences that depression played a mediating role in the association between PIS and suicide. The indirect effect of PIS on suicide through depression was as important as the direct effect. Interventions focused on reducing PIS of HIV/AIDS and depressive symptoms of PLWH simultaneously may be effective in reducing suicidal ideation and attempts among PLWH.

## Abbreviations

AIDS: Acquired immune deficiency syndrome
ART: Antiretroviral therapy
CDCs: Centers for disease control and prevention
CES-D: Center for epidemiologic studies depression scale
CFA: Confirmatory factor analysis
CFI: Comparative fit index
DA: Depressed affect
DF: Degree of freedom
FSWs: Female sex workers
HIV: Human immunodeficiency virus
IP: Interpersonal problems
IQR: Interquartile range
IS: Internalized stigma
MLR: Robust maximum likelihood
MSM: Men who have sex with men
NGOs: Non-governmental organizations
PA: Positive affect
PIS: Perceived and internalized stigma
PLWH: People living with HIV/AIDS
PS: Perceived stigma
RMSEA: Root mean square error of approximation
SD: Standard deviation
SEM: Structural equation model
SRA: Somatic and retarded activity
SRMR: Standardized root mean square residual
TLI: Tucker-Lewis index
WLS: Weighted least squares
WRMR: Weighted root mean square residual
